# Identification and characterization of bone/cartilage-associated signatures in common fibrotic skin diseases

**DOI:** 10.3389/fgene.2023.1121728

**Published:** 2023-04-04

**Authors:** Ting Wu, Yifan Jin, Fangqi Chen, Xiuyun Xuan, Juanmei Cao, Yan Liang, Yuqing Wang, Jinshan Zhan, Mengjie Zhao, Changzheng Huang

**Affiliations:** ^1^ Department of Dermatology, Union Hospital, Tongji Medical College, Huazhong University of Science and Technology, Wuhan, China; ^2^ Department of Dermatology, Zhongnan Hospital of Wuhan University, Wuhan, China

**Keywords:** fibrotic skin disease, keloid, systemic sclerosis, bone/cartilage-related gene, bioinformatics analysis

## Abstract

**Background:** Fibrotic skin diseases are characterized by excessive accumulation of the extracellular matrix (ECM) and activation of fibroblasts, leading to a global healthcare burden. However, effective treatments of fibrotic skin diseases remain limited, and their pathological mechanisms require further investigation. This study aims to investigate the common biomarkers and therapeutic targets in two major fibrotic skin diseases, namely, keloid and systemic sclerosis (SSc), by bioinformatics analysis.

**Methods:** The keloid (GSE92566) and SSc (GSE95065) datasets were downloaded from the Gene Expression Omnibus (GEO) database. Differentially expressed genes (DEGs) were identified, followed by functional enrichment analysis using Gene Ontology (GO) and Kyoto Encyclopedia of Genes and Genomes (KEGG). We then constructed a protein–protein interaction (PPI) network for the identification of hub genes. We explored the possibility of further functional enrichment analysis of hub genes on the Metascape, GeneMANIA, and TissueNexus platforms. Transcription factor (TF)–hub gene and miRNA–hub gene networks were established using NetworkAnalyst. We fixed GSE90051 and GSE76855 as the external validation datasets. Student’s t-test and receiver operating characteristic (ROC) curve were used for candidate hub gene validation. Hub gene expression was assessed *in vitro* by quantitative real-time PCR.

**Results:** A total of 157 overlapping DEGs (ODEGs) were retrieved from the GSE92566 and GSE95065 datasets, and five hub genes (COL11A1, COL5A2, ASPN, COL10A1, and COMP) were identified and validated. Functional studies revealed that hub genes were predominantly enriched in bone/cartilage-related and collagen-related processes. FOXC1 and miR-335-5p were predicted to be master regulators at both transcriptional and post*‐*transcriptional levels.

**Conclusion:** COL11A1, COL5A2, ASPN, COL10A1, and COMP may help understand the pathological mechanism of the major fibrotic skin diseases; moreover, FOXC1 and miR-355-5p could build a regulatory network in keloid and SSc.

## Introduction

Fibrosis is characterized by fibroblast proliferation and excessive extracellular matrix deposition in the dermis, which contributes to significant morbidity and mortality (Deng et al., 2021). Fibrotic skin diseases, such as keloid and scleroderma, impose a significant physiological and psychological burden (Nietert et al., 2005; Lv et al., 2020); however, the pathogenesis of the two diseases has to be elucidated yet, and effective therapeutics are currently unavailable (Lee and Pope, 2016; Limandjaja et al., 2020). Previous studies have found similarities and interactions in fibrosis molecular mechanisms, such as the activation of the Notch signaling cascade and TGF-β pathway (Condorelli et al., 2021), shared regulators of miRNA-21 (Li et al., 2017) and miRNA-29 (Deng et al., 2017), and active association between keratinocytes and fibroblasts (Russo et al., 2020). Therefore, screening differentially expressed genes between keloid and SSc could provide an alternative route to identify the mechanisms involved in the fibrosis process.

In recent years, the validation of biomarker expression has been facilitated with the increasing use of tissue microarrays (Hassan et al., 2008). COMP (Agarwal et al., 2013), POSTN (Deng et al., 2021), KGF (Canady et al., 2013), and MMP (Kelly et al., 2010), for example, have been shown to play important roles in ECM deposition and myofibroblast differentiation. The functional role of miRNAs, such as miR-29a-3p (He et al., 2022) and miR-16-5p (Yao et al., 2020), in the progression of keloid and SSc, has been demonstrated through the integration of bioinformatics technology and clinical treatment. CircRNAs have been investigated in other fibrosing processes. Circ_0070963, circRNA_010567, and circ_0044226 have been confirmed as potential therapeutic targets for liver fibrosis (Ji et al., 2020), myocardial fibrosis (Zhou and Yu, 2017), and pulmonary fibrosis (Qi et al., 2020), respectively. However, bioinformatics analysis of fibrotic skin diseases has rarely been performed. In this study, we collected and analyzed RNA sequence data from keloid and SSc, with an aim to shed light on their pathogenesis and provide a potential treatment direction.

## Materials and methods

### Data collection

The Gene Expression Omnibus ( http://www.ncbi.nlm.nih.gov/geo/) is an open international public database for next-generation functional genomic sequence datasets and high-throughput microarrays (Barrett et al., 2013). We extracted two datasets from GEO, namely, GSE92566, including four keloid and three non-lesional skin samples, and GSE95065, incorporating 18 samples of diffuse cutaneous systemic sclerosis and 15 samples of normal skin. GSE92566 and GSE95065 microarrays were obtained using an Affymetrix Human Genome U133 Plus 2.0 Array and Affymetrix Human Genome U133A 2.0 Array (HGU133A2 Hs ENTREZG 19.0.0), respectively.

### Identification of DEGs

GEO2R (http://www.ncbi.nlm.nih.gov/geo/geo2r/) is an interactive tool based on limma algorithm that assists users with GEO data analysis (Davis and Meltzer, 2007). | log (fold change) | > 1 and a *p*-value < 0.05 were considered statistically significant parameters. Then, the volcano plot was created on a web platform (https://www.bioinformatics.com.cn). A Venn diagram was generated using Rtools 4.2 to reveal ODEGs.

### Gene Ontology and Kyoto Encyclopedia of Genes and Genomes pathway analyses

GO and KEGG analyses were carried out using the Database for Annotation, Visualization and Integrated Discovery (DAVID) tool (https://david.ncifcrf.gov/), which was used for the functional annotation of DEGs and summarization of the relevant biological patterns (Huang et al., 2009). GO consisted of biological processes (BPs), cellular components (CCs), and molecular functions (MFs). A two-tailed *p*-value < 0.05 was set as the threshold of statistical significance.

### Protein–protein interaction network construction

The protein–protein interaction network was predicted by the Search Tool for the Retrieval of Interacting Genes (STRING, version 11.0, https://string-db.org/) with a combined score > 0.4 (medium confidence). The PPI network was then analyzed in Cytoscape software (version 3.8.0), and the Molecular Complex Detection plugin (MCODE, version 2.0.0) was used to find the most significant module with the following parameters: degree cut-off = 2, node score cut-off = 0.2, k-score = 2, and max depth = 100. The top 10 genes were filtered for further analysis on the cytoHubba platform using maximal clique centrality (MCC) and edge percolated component (EPC) algorithms. Heatmaps were created to assess the two‐way hierarchical clustering analysis of the hub genes in two datasets using Rtools 4.2.

### Functional enrichment analysis and annotation of hub genes

To further explore the biological process (BP) of hub genes, DAVID was used to analyze them again. Metascape is intended to provide comprehensive gene list annotation and deconstruct the molecular mechanisms underlying a biological system (Zhou et al., 2019). The five hub genes were submitted to the Metascape online tool for GO cluster analysis. To perform enrichment analysis and related functional annotations of target genes, genes were divided into clusters using terms with a *p*-value of 0.01, a minimum count of 3, and an enrichment factor > 1.5. GeneMANIA is used to predict the relative interactive genes of hub genes and to integrate co-expression networks for these genes (Warde-Farley et al., 2010). GeneMANIA’s network consists of physical interactions, co-expression, predicted, co-localization, genetic interactions, pathway, and shared protein domains. TissueNexus is a database of 49 human tissue/cell line functional gene networks that can be used for network search, visualization, and functional analysis (Lin C. X et al., 2022). We further utilized the database to construct a network of functional annotations of the hub genes.

### Construction of the regulation network of transcription factors (TFs) and miRNAs

TFs and miRNAs of the hub genes were predicted *via* NetworkAnalyst. The relevant TFs were obtained from the JASPAR database, and the miRNAs were acquired from miRTarBase v8.0 and TarBase v8.0 (Zheng et al., 2022). Then, the network of TFs and miRNAs was, respectively, constructed using Cytoscape 3.8.2 software.

### Tissue samples

After the patients signed the informed consent, three cases of human normal tissues, keloid tissues, and SSc tissues were obtained from the Department of Dermatology, Union Hospital, Tongji Medical College (Wuhan, China). Then, they were stored at 4°C for total RNA isolation. The clinical information is shown in Table 1.

**TABLE 1:** Clinical information of validated samples.

Sample type	Sample location	Gender	Age (year)
Keloid 1	Trunk	Female	25
Keloid 2	Trunk	Female	44
Keloid 3	Upper extremity	Male	27
SSc 1	Trunk	Female	38
SSc 2	Trunk	Male	50
SSc 3	Trunk	Male	29
Normal tissue	Trunk	Female	22
Normal tissue	Trunk	Male	36
Normal tissue	Foreskin	Male	20

### Validation of the performance of hub genes

The expression of the hub genes in keloid, SSc, and normal tissues was validated using Student’s *t*‐test in GraphPad Prism 8.0.1, and their specificity and sensitivity were further evaluated by the receiver operating characteristic curve analysis.

The total RNA was isolated using TRIzol (Vazyme, China) according to the manufacturer’s instructions. Approximately 1 mg of the total RNA from each sample was reverse-transcribed into cDNA using the PrimeScriptTM RT Master Mix kit (Takara, RR036A). Real-time PCR was performed using the TB Green Premix Ex TaqTM kit (Takara, RR420A) according to the manufacturer’s instructions. The primers for human were as follows: COMP: forward 5′-CAT​CAG​GAC​TCT​CGG​GAC​AAC​T-3′ and reverse 5′-GTC​TAC​CAC​CTT​GTC​TGC​ATC​AA-3′; ASPN: forward 5′- AGT​CCC​AAC​CAA​CAT​TCC​ATT​TG-3′ and reverse 5′- TGG​GTG​AAT​CTT​CGT​TAG​CTT​GT-3′; COL5A2: forward 5′-GAA​GCC​TCC​CAG​AAC​ATC​ACT​TA-3′ and reverse 5′-CCC​ACA​TTT​CCA​TTC​CGC​TTA​GA-3′; COL10A1: forward 5′-GCA​TAA​AAG​GCC​CAC​TAC​CCA-3′ and reverse 5′- GCA​TAA​AAG​GCC​CAC​TAC​CCA-3′; COL11A1: forward 5′- AAG​ACG​GAG​ACA​AGG​GTG​AAA​TT-3′ and reverse 5′- TTC​ACC​ATC​ACC​TCC​AGC​AAT​TC-3′; and GAPDH: forward 5′-GGA​GTC​CAC​TGG​CGT​CTT​CA-3′ and reverse 5′-GTC​ATG​AGT​CCT​TCC​ACG​ATA​CC-3′. The relative mRNA expression level was standardized to GAPDH and was calculated using the 2^−ΔΔCT^ method. Statistically significant differences are indicated by *— *p < 0.05, **p < 0.01, and ***p < 0.001.

In addition, GSE90051 was used to validate the expression of hub genes in keloids and adjacent normal tissues, while GSE76885 was used to verify the expression pattern of hub genes in SSc and normal tissues.

## Results

### DEG identification

**FIGURE 1:**
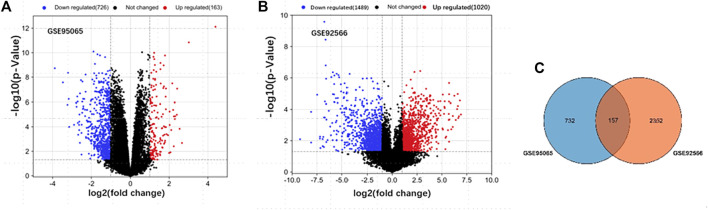
**(A, B)** Volcano plots of the DEGs in GSE95065 and GSE92566 datasets. Red and blue represent the upregulated and downregulated genes, respectively. **(C)** Venn diagram of the ODEGs in the two datasets.

After standardization, a total of 889 DEGs (163 upregulated and 726 downregulated) with SSc in GSE95065 and 2,509 DEGs (1,020 upregulated and 1,489 downregulated) with keloids in GSE92566 were identified (Figures 1A, B). There were 157 ODEGs between GSE95065 and GSE92566, as shown in the Venn diagram (Figure 1C), including 56 consistently upregulated DEGs and 77 downregulated DEGs.

### GO and KEGG enrichment analyses of ODEGs

**FIGURE 2:**
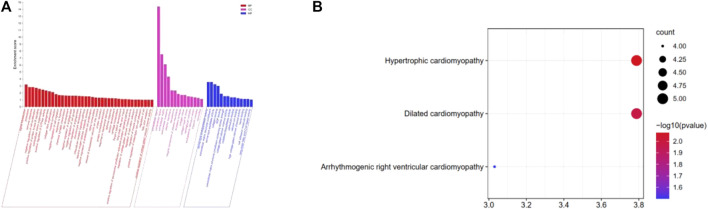
**(A)** GO analysis of the ODEGs. **(B)** KEGG pathway enrichment analysis of the ODEGs.

Using the *p*-value < 0.05 criterion, we identified the significant GO enrichment of ODEGs (Figure 2A). Among the upregulated ODEGs, BPs were highly associated with collagen fibril organization, immune response, and extracellular matrix organization. The CC was involved in the extracellular region, extracellular space, and extracellular matrix. Top MF was significantly enriched in extracellular matrix structural constituents, collagen binding, and metalloendopeptidase activity. Among the downregulated ODEGs, BP was mostly enriched in muscle contraction, positive regulation of protein kinase B signaling, and long-chain fatty acid import; CC was mostly enriched in extracellular exosome, extracellular region, and sarcolemma; and MF was mostly enriched in serine-type peptidase activity, serine-type endopeptidase activity, and protein binding. Furthermore, KEGG pathway analysis (Figure 2B) revealed that the upregulated ODEGs were involved in protein digestion and absorption and the downregulated ODEGs in hypertrophic cardiomyopathy, dilated cardiomyopathy, and arrhythmogenic right ventricular cardiomyopathy.

### Protein–protein interaction network construction and hub gene analysis

**FIGURE 3:**
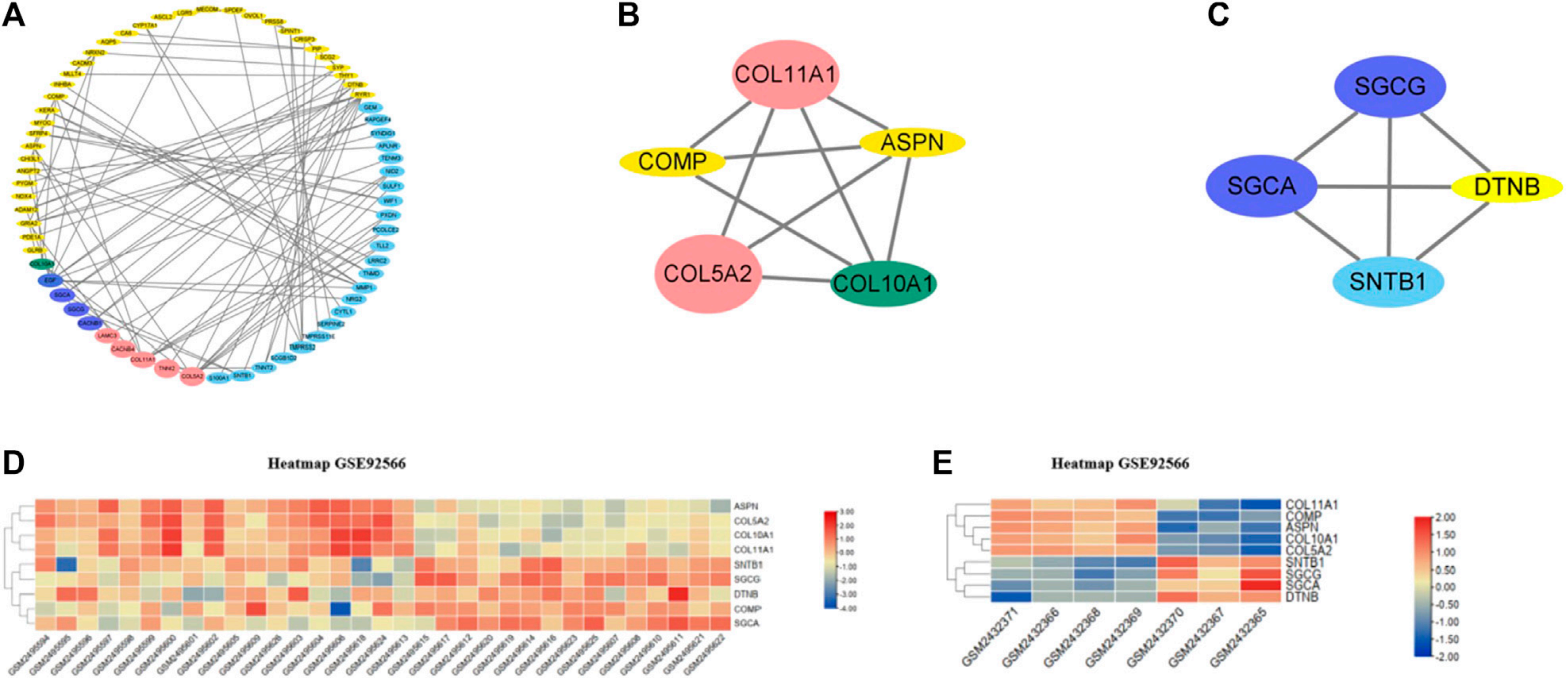
**(A)** PPI networks of ODEGs constructed by Cytoscape. **(B–C)** Main modules obtained using MCODE. **(D–E)** Heatmaps of the hub genes in GSE92566 and GSE95065 datasets. Red and blue represent upregulation and downregulation of expression, respectively.

A total of 133 consistently changed ODEGs were placed in STRING to explore the interactive networks with free proteins removed. Cytoscape was used to visualize PPI, which consisted of 67 nodes and 94 edges (Figure 3A). Based on maximal clique centrality (MCC) and edge percolated component (EPC) algorithms, the top 10 genes were identified using cytoHubba (Table 2). COLLA1, COL5A2, ASPN, COL10A1, EGF, COMP, and SYP were identified as key genes. Then, MCODE algorithm was utilized to reveal the most significant modules, as shown in Figures 3B, C. Module1 (Figure 3B) contained five upregulated DEGs (COL11A1, COL5A2, COL10A1, ASPN, and COMP); module 2 (Figure 3C) consisted of four downregulated DEGs (SGCG, SGCA, SNTB1, and DTNB). As a result of the overlap of cytoHubba and MCODE, COMP, ASPN, COL10A1, COL11A1, and COL5A2 were identified as hub genes. Then, heatmaps (Figures 3D, E) were constructed, which revealed a high degree of clustering of these hub genes.

**TABLE 2:** Top 10 genes in the network using MCC and EPC algorithms.

Algorithm	Top 10 genes in the network
MCC	COL11A1, COL5A2, ASPN, COL10A1, EGF, RYR1, and COMP
EPC	EGF, THY1, SYP, NRXN2, COL5A2, COL11A1, and COMP

### Enrichment analysis and functional annotation of hub genes

**FIGURE 4:**
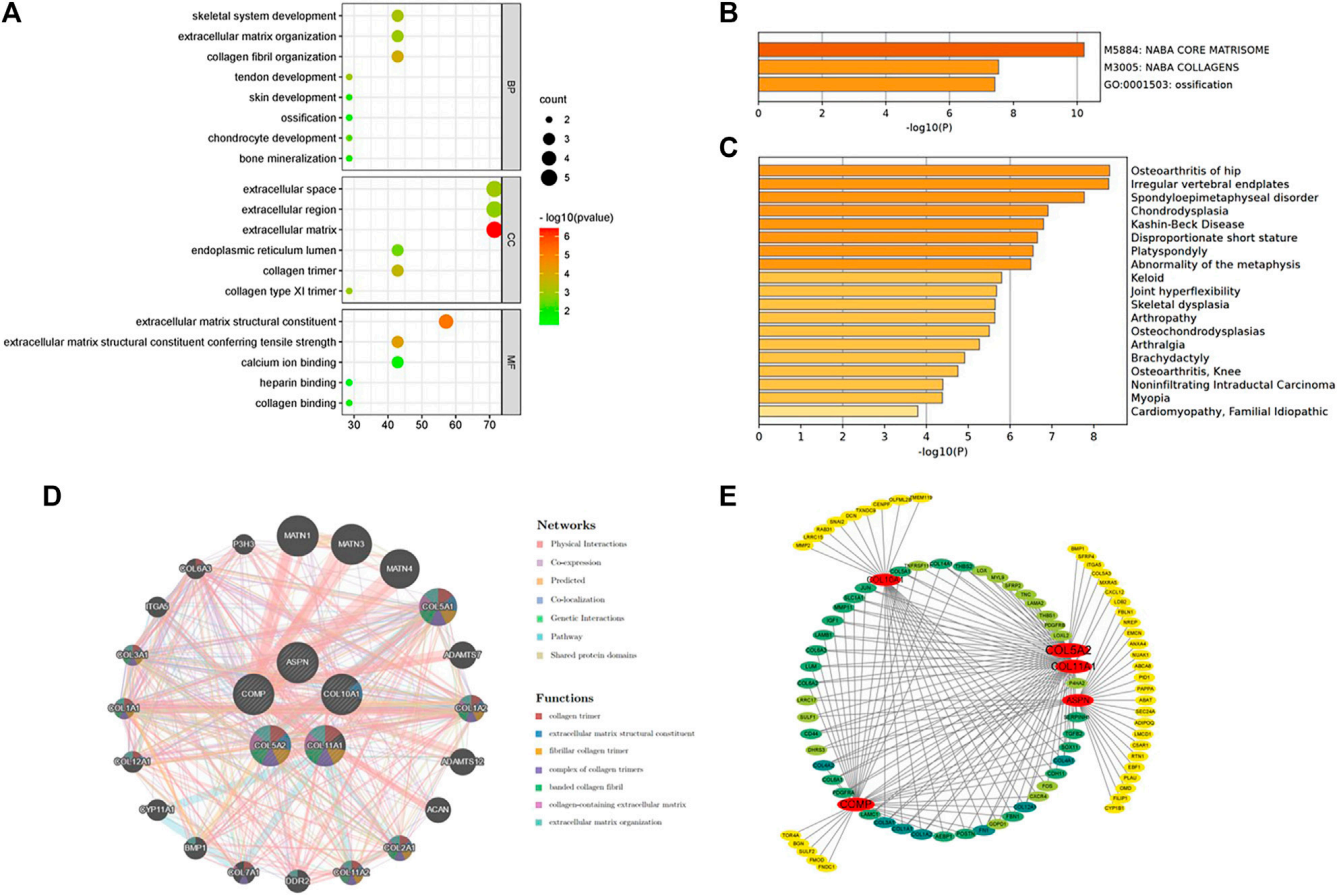
**(A)** Biological processes enriched by the five hub genes. **(B)** Pathway and process enrichment analysis on the Metascape platform. **(C)** Enrichment analysis in DisGeNET. **(D)** Interaction network of the hub genes and their predicted genes *via* GeneMANIA. **(E)** Functional annotation of hub genes using TissueNexus.

The five hub genes were entered into DAVID for GO analysis. BPs of these genes were most prominent in collagen fibril organization, skeletal system development, and tendon or chondrocyte development (Figure 4A).

The Metascape platform summarized the functional enrichment analysis of the hub genes. The bar graph (Figure 4B) showed that these genes mainly enriched in naba core matrisome, naba collagens, and ossification. Interestingly, DisGeNET (Figure 4C) also revealed that the hub genes were primarily enriched in bone/cartilage-related diseases such as osteoarthritis of the hip, irregular vertebral endplates, spondyloepimetaphyseal disorder, and chondrodysplasia. It could shed light on the possible pathogenesis of fibrotic skin disorders.

GeneMANIA was used to create a protein interaction network of the five hub genes and the 20 predicted genes (Figure 4D). Physical interactions (77.64%), co-expression (8.01%), predicted (5.37%), co-localization (3.63%), genetic interactions (2.87%), pathways (1.88%), and shared protein domains (0.60%) are among the networks based on GeneMANIA’s functional annotation patterns. The function of predicted genes was mostly associated with collagen formation (Table 3). It is worth noting that there were proteins related to skeletal system development (six of the predicted genes) and regulation of cartilage development (three of the predicted genes).

**TABLE 3:** Gene functions analyzed using GeneMANIA.

Function	FDR	Genes in the network	Genes in the genome
Collagen trimer	4.30E-22	11	28
Extracellular matrix structural constituent	4.69E-19	11	51
Fibrillar collagen trimer	5.67E-19	8	10
Complex of collagen trimers	7.50E-19	9	19
Banded collagen fibril	1.25E-18	8	11
Collagen-containing extracellular matrix	6.94E-15	10	80
Extracellular matrix organization	1.07E-13	11	164
Endoderm formation	2.74E-08	6	42
Endoderm development	8.29E-08	6	51
Formation of the primary germ layer	8.87E-07	6	76
Gastrulation	7.67723E-06	6	110
Embryonic morphogenesis	0.000100714	7	296
Growth factor binding	0.000184246	5	96
Skeletal system development	0.000221816	6	201
Connective tissue development	0.00042998	5	117
Sensory perception of the mechanical stimulus	0.007941999	4	97
Regulation of cartilage development	0.01332598	3	35
Collagen metabolic process	0.089683105	3	67

TissueNexus was used to create functional gene networks. COL5A2 was discovered to be the most important player in the network (Figure 4E). Furthermore, the top three functional annotations of the hub genes were extracellular matrix organization, extracellular structure, and external encapsulating structure (Table 4). The functional annotation further demonstrated that bone/cartilage-related genes participated in the process of extracellular matrix deposition.

**TABLE 4:** Functional annotation of hub genes using TissueNexus.

GO ID	GO name	FDR
GO:0030198	Extracellular matrix organization	2.24E-34
GO:0043062	Extracellular structure organization	2.52E-34
GO:0045229	External encapsulating structure organization	3.17E-34
GO:0030199	Collagen fibril organization	4.55E-31
GO:0001503	Ossification	6.59E-13
GO:0042060	Wound healing	2.05E-12
GO:0035987	Endodermal cell differentiation	3.8E-11
GO:0071559	Response to transforming growth factor beta	1.52E-10
GO:0061448	Connective tissue development	2.22E-10
GO:0001706	Endoderm formation	2.75E-10

### Analyses of the interactions of TF–hub genes and miRNA–hub genes

**FIGURE 5:**
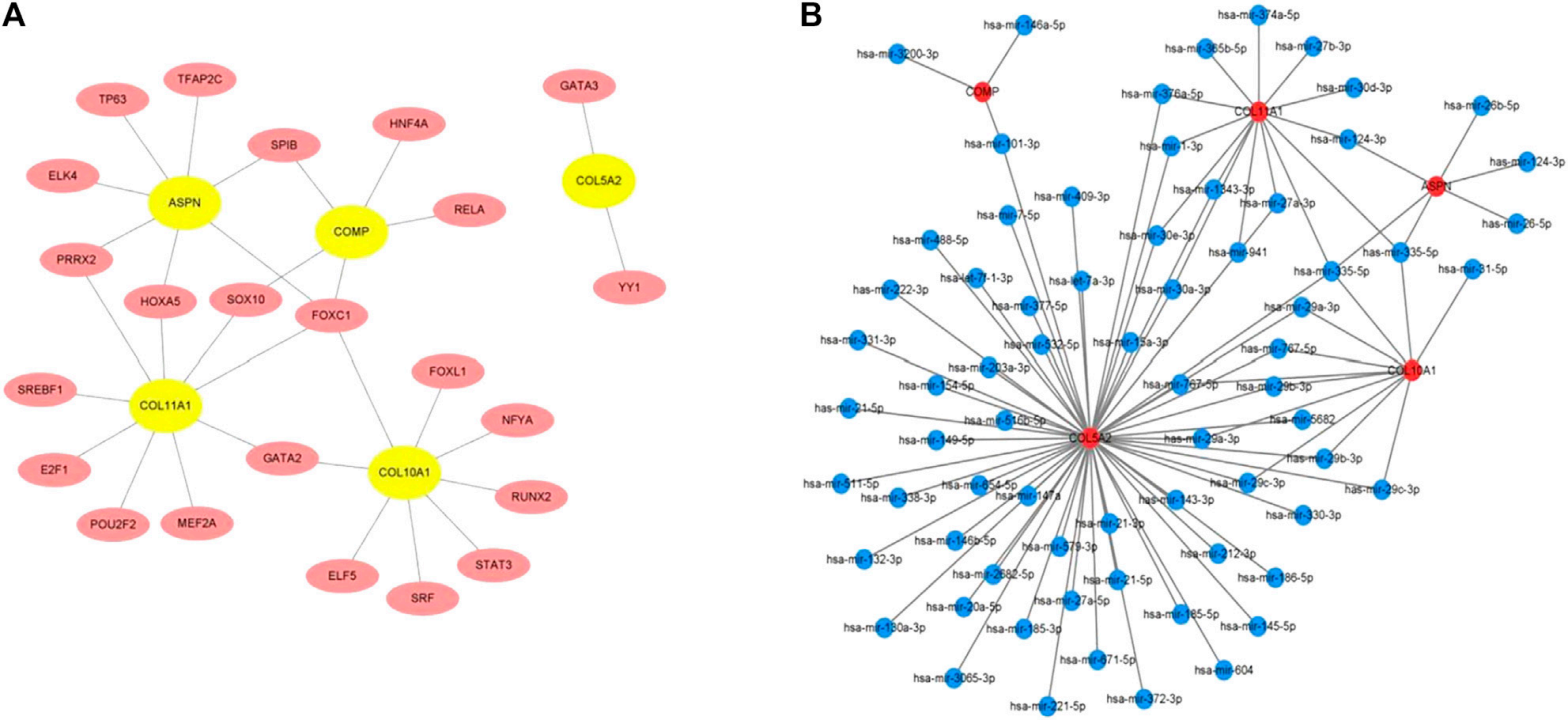
**(A)** TF–hub gene network established using the JASPAR database on NetworkAnalyst. **(B)** miRNA–hub gene network acquired utilizing miRTarBase v8.0 and TarBase v8.0 *via* NetworkAnalyst.

The TFs of hub genes were acquired using the JASPAR database on the NetworkAnalyst web tool. The miRNAs of the hub genes were obtained using miRTarBase v8.0 and the TarBase v8.0 *via* NetworkAnalyst. The regulatory network between five seeds (COMP, ASPN, COL5A2, COL10A1, and COL11A1) and their TFs was established using Cytoscape (Figure 5A). The TF–hub genes had 28 nodes and 31 edges. FOXC1 may play an important role in the network because it has the potential to regulate COL5A2, COL11A1, ASPN, and COL10A1 simultaneously. The miRNA–hub gene regulatory network had 75 nodes and 92 edges (Figure 5B). Has-miR-335-5p was most likely targeted by COL5A2, COL11A1, ASPN, and COL10A1.

### Validation of hub genes

**FIGURE 6:**
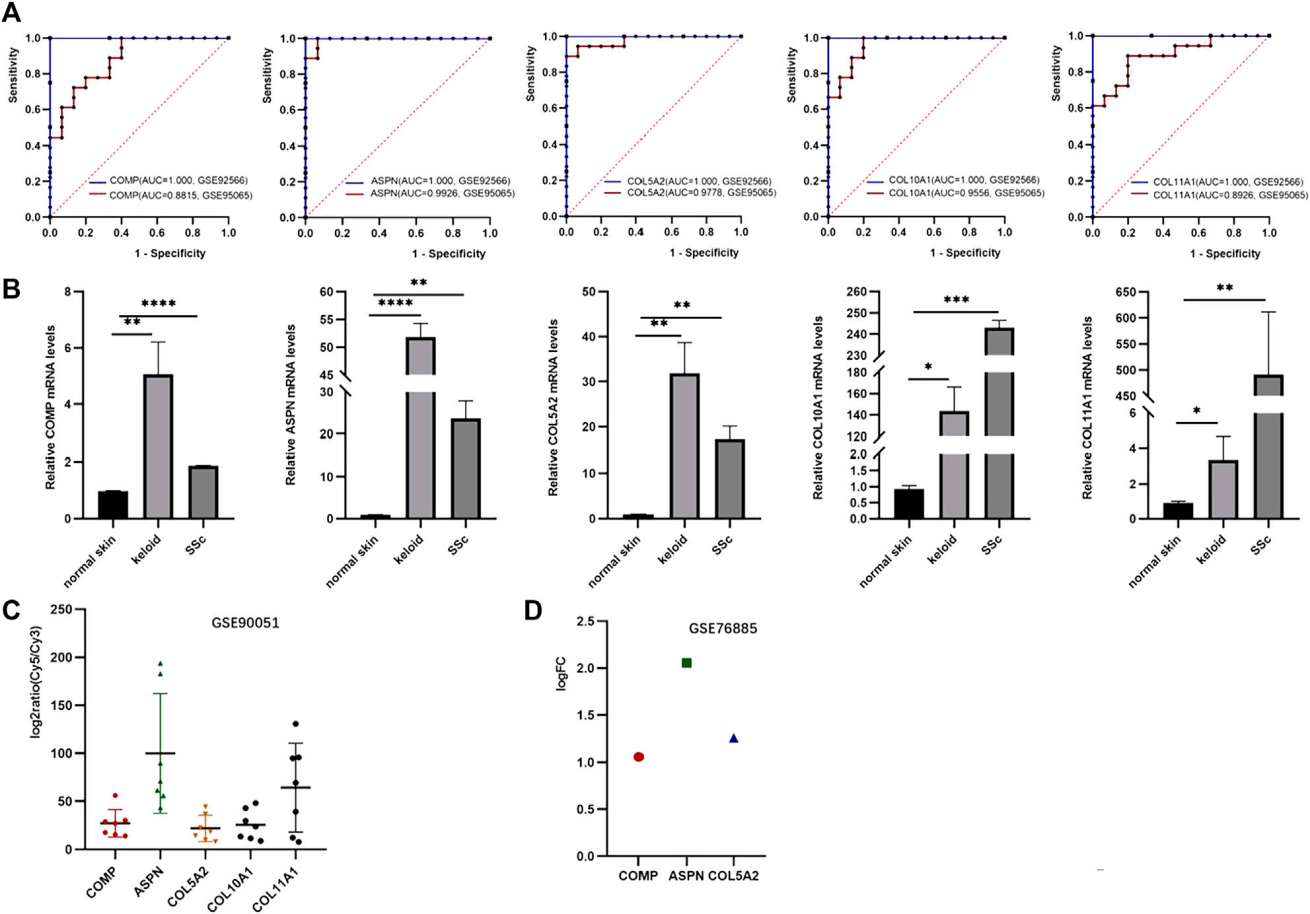
Validation of hub genes. **(A)** ROC curves demonstrating the diagnostic value of the hub genes. **(B)** Expression level of hub genes validated by qPCR. **(C)** Hub gene validation in the GSE90051 dataset. **(D)** Hub gene validation in the GSE76855 dataset.

The expression of five hub genes was higher in group keloid and SSc tissues than in normal tissues (*p* < 0.05). Furthermore, the AUC values of the ROC curves (Figure 6A) for the five genes were all greater than 0.8, indicating high sensitivity and specificity. The hub genes (COMP, ASPN, COL10A1, COL11A1, and COL5A2) were found to be reliable biomarkers with high diagnostic accuracy (Supplementary Table S1). We validated the expression of hub genes by qRT-PCR in clinical samples (Figure 6B). Compared to normal tissues, these genes were significantly highly expressed in keloid and SSc. In addition, the hub genes were then validated using external datasets. Notably, GSE90051 described familiar changes in the hub genes, which were more abundant in keloid tissues than in normal tissues (Figure 6C). As for the dataset GSE76855, we only disclosed the expression of three hub genes (COMP, ASPN, and COL5A2). Thankfully, COMP, ASPN, and COL5A2 were all upregulated in SSc tissues compared to normal tissues, supporting our findings (Figure 6D).

## Discussion

Fibrotic skin diseases including keloid, hypertrophic scar, systemic sclerosis, and lichen sclerosus et atrophicus manifest analogous histopathology but unclear etiopathogenesis. Considering the reported relationships between keloid and SSc, screening biomarkers associated with fibrosis is a promising way to gain insights into common fibrotic skin diseases at a genomic level. To the best of our knowledge, no similar study has been reported on the common biomarkers in keloid and SSc by bioinformatics analysis, which could contribute to discovering underlying mechanisms of cutaneous fibrosis and novel therapeutic targets.

A total of 157 ODEGs obtained from keloid (GSE92566) and SSc (GSE95065) datasets were found to be related to cardiomyopathy pathways by KEGG and extracellular components/processes by GO analysis, respectively. A total of five hub genes (COMP, ASPN, COL5A2, COL10A1, and COL11A1) were identified by MCC, EPC, and MCODE algorithms relying on STRING and Cytoscape platforms. Surprisingly, the filtered hub genes were also closely associated with the excess production of bone and cartilage proteins in the extracellular matrix of fibrotic skin diseases, according to the functional enrichments.

COMP encodes cartilage oligomeric matrix protein, an extracellular matrix glycoprotein found in cartilage, tendons, ligaments, and the growth plate (Hedbom et al., 1992). Mutations in the COMP genes cause pseudoachondroplasia (PSACH) and multiple epiphyseal dysplasia (MED/EDM1) (Briggs et al., 1995). COMP has been reported to affect the secretion of type IX collagen and matrilin-3 (Hecht et al., 2005) and to bind to collagen types I and II (Södersten et al., 2005) and fibronectin (Di Cesare et al., 2002), thereby maintaining an ECM network. In clinical practice, COMP is used as a prognostic marker for joint injury, a biomarker for idiopathic pulmonary fibrosis, and a biomarker for cartilage degeneration-associated osteoarthritis and rheumatoid arthritis (Posey et al., 2018). Recently, pathogenesis of various fibrotic diseases, such as liver fibrosis (Magdaleno et al., 2016) and renal fibrosis (Kim et al., 2006), has been linked to COMP deposition. In line with previous research (Farina et al., 2006; Inui et al., 2011; Agarwal et al., 2013), our study also supported that COMP overexpression is a common feature of fibrotic skin diseases.

ASPN, a member of the small leucine-rich proteoglycan (SLRP) family of extracellular proteins, was initially discovered in human cartilage (Lorenzo et al., 2001). Copy number loss in the ASPN gene region may be involved in the pathogenesis of acetabular dysplasia (Sekimoto et al., 2017). ASPN has been identified as a novel mesenchymal stromal cell factor that affects tumor microenvironment and metastatic progression (Hughes et al., 2019), including prostate cancer (Hurley et al., 2016), breast cancer (Maris et al., 2015), colorectal cancer (Huang C. Z et al., 2022), and others. It also played a crucial biological role in the process of tissue fibrosis (Liu et al., 2021; Huang S. et al., 2022; Huang C. et al., 2022). Huang S. et al. (2022) reported that ASPN could promote lung myofibroblast differentiation by facilitating TGF-β/Smad signaling. Huang C. et al. (2022) unveiled that ASPN could attenuate fibrosis in cardiac remodeling by regulating mitochondrial bioenergetics and protecting cardiomyocytes from hypoxia–reoxygenation-mediated cell death. Liu et al. (2021) showed that ASPN participated in keloid progression by disturbing interfibroblast mechanocommunication and inhibiting matrix remodeling.

COL5A2, COL10A1, and COL11A1 are members of the collagen gene family that are highly expressed in bone/cartilage-associated diseases (Gu et al., 2014; Yang et al., 2018; Sun et al., 2020). In the early stage of osteoblast differentiation, COL5A2 may help with bone material (Tawa et al., 2021) and actin skeleton formation (Hong et al., 2010). Mutations in COL10A1, which is a specific marker of hypertrophic chondrocytes (Gu et al., 2014), result in Schmid metaphyseal chondrodysplasia (SMCD), an autosomal dominantly inherited skeletal disorder (Bateman et al., 2005). COL11A1 is a fibril-forming collagen that regulates cartilage and tendon fibrillar structure (Sun et al., 2020). Stickler syndrome type II is caused by COL11A1 gene mutations (Vogiatzi et al., 2018). Previous bioinformatics analysis has demonstrated that COL5A2, COL10A1, and COL11A1 may be used as a diagnostic or prognostic biomarker for tumors such as gastric cancer (Niu et al., 2022), breast carcinoma (Giussani et al., 2018), and non-small-cell lung cancer (Andriani et al., 2018). According to the findings of RNA sequencing, COL5A2, COL10A1, and COL11A1 have been reported to be upregulated in keloid (Bi et al., 2021; Lin P. et al., 2022), as in our study. A whole-exome sequencing study revealed that COL5A2 is relevant to the fibrotic features of diffuse cutaneous SSc (Mak et al., 2016). However, the relationship between these genes and SSc is poorly understood.

In recent years, we have considered that fibroblasts differ in their heterogeneity depending on their anatomical location (Lynch and Watt, 2018). Human skin single-cell transcriptomes indicated that fibroblasts are classified into four subpopulations: secretory-papillary, secretory-reticular, mesenchymal, and pro-inflammatory (Solé-Boldo et al., 2020). Interestingly, the upregulated genes like COMP, COL5A2, and COL11A1 were enriched in mesenchymal fibroblasts reported by single-cell RNA-seq (Deng et al., 2021). Thus, we hypothesized that mesenchymal fibroblasts secrete bone and cartilage proteins, which contribute to fibrosis.

In our study, the transcription factor (FOXC1) and miRNA (miR-335-5p) were predicted to be the key transcriptional and post-transcriptional regulators of DEGs. FOXC1 has been linked to cartilage differentiation (Xu et al., 2021), vascular development (Shu et al., 2022), tumor development, and metastasis (Zhang et al., 2020; Lin Z. et al., 2021). High miR-335-5p expression promotes bone formation and regeneration (Zhang et al., 2017) and angiogenesis (Walz et al., 2019) and regulates cancer progression by targeting MAPK10 (Gao et al., 2021) or NLRP1/7 (Lin W. et al., 2021). Furthermore, a FOXC1 and miR-335-5p network was presented in non-small-cell lung cancer (Mosharaf et al., 2022), type 2 diabetes (Rahman et al., 2020), and vascular dementia (Shu et al., 2022). The dermis of keloid and SSc is known to be made up of newly formed vessels (Trojanowska, 2010; Ogawa, 2017). The FOXC1 and miR-335-5p networks may collaborate in the keloid and SSc processes. This is the first study to look into the relationship between FOXC1 and miR-335-5p in fibrotic skin diseases.

In summary, functional enrichments showed that bone/cartilage-associated genes are overexpressed in keloid and SSc. After validation, the five hub genes can be identified as diagnostic biomarkers of fibrotic skin diseases.

## Conclusion

In our present study, we screened bone/cartilage-related genes (COMP, ASPN, COL5A2, COL10A1, and COL11A1), transcription factor (FOXC1), and miRNA (miR-335-5p) in major fibrotic skin diseases including keloid and SSc. We hope to provide further group work and insights into fibrotic skin diseases and find potential therapeutic targets for clinical treatments.

## Data Availability

The original contributions presented in the study are included in the article/Supplementary Material; further inquiries can be directed to the corresponding authors.
