# Supplementary material for: Identification and characterization of bone/cartilage-associated signatures in common fibrotic skin diseases
Source: Front Genet. 2023 Apr 4;14:1121728. doi: 10.3389/fgene.2023.1121728 (PMC10111020; doi:10.3389/fgene.2023.1121728)
Supplement: Supplementary file 1 [file DataSheet1.docx]

Supplementary Table 1 The 95% confidence interval and P value of ROC curve.

|  | GSE 92566 | | | | | GSE95065 | | | | |
| --- | --- | --- | --- | --- | --- | --- | --- | --- | --- | --- |
|  | COMP | ASPN | COL5A2 | COL10A1 | COL11A1 | COMP | ASPN | COL5A2 | COL10A1 | COL11A1 |
| 95% CI | 1.000 to 1.000 | 1.000 to  1.00 | 1.000  to  1.000 | 1.000  to  1.00 | 1.000  to  1.00 | 0.7687 to 0.9943 | 0.9732 to  1.000 | 0.9357  to  1.000 | 0.8931  to  1.000 | 0.7849  to  1.000 |
| P value | 0.0339 | 0.0339 | 0.0339 | 0.0339 | 0.0339 | 0.0002 | <0.0001 | <0.0001 | <0.0001 | 0.0001 |
